# Inhibition of NO Biosynthetic Activities during Rehydration of *Ramalina farinacea* Lichen Thalli Provokes Increases in Lipid Peroxidation

**DOI:** 10.3390/plants8070189

**Published:** 2019-06-26

**Authors:** Joana R. Expósito, Sara Martín San Román, Eva Barreno, José Reig-Armiñana, Francisco José García-Breijo, Myriam Catalá

**Affiliations:** 1 Department of Biology and Geology, Physics and Inorganic Chemistry, ESCET—Campus de Móstoles, C/Tulipán s/n, E-28933 Móstoles (Madrid), Spain; 2 Universitat de València, Botánica & ICBIBE—Jardí Botànic, Fac. CC. Biológicas, C/Dr. Moliner 50, 46100 Burjassot, Valencia, Spain; 3 U. Politècnica de València, Dpto. Ecosistemas Agroforestales, Camino de Vera s/n, 46020 Valencia, Spain

**Keywords:** *Trebouxia*, microalgae, lipid peroxidation, diaphorase activity, lichens, nitric oxide, nitrate reductase, nitric oxide synthase

## Abstract

Lichens are poikilohydrous symbiotic associations between a fungus, photosynthetic partners, and bacteria. They are tolerant to repeated desiccation/rehydration cycles and adapted to anhydrobiosis. Nitric oxide (NO) is a keystone for stress tolerance of lichens; during lichen rehydration, NO limits free radicals and lipid peroxidation but no data on the mechanisms of its synthesis exist. The aim of this work is to characterize the synthesis of NO in the lichen *Ramalina farinacea* using inhibitors of nitrate reductase (NR) and nitric oxide synthase (NOS), tungstate, and NG-nitro-L-arginine methyl ester (L-NAME), respectively. Tungstate suppressed the NO level in the lichen and caused an increase in malondialdehyde during rehydration in the hyphae of cortex and in phycobionts, suggesting that a plant-like NR is involved in the NO production. Specific activity of NR in *R. farinacea* was 91 μU/mg protein, a level comparable to those in the bryophyte *Physcomitrella patens* and *Arabidopsis thaliana*. L-NAME treatment did not suppress the NO level in the lichens. On the other hand, NADPH-diaphorase activity cytochemistry showed a possible presence of a NOS-like activity in the microalgae where it is associated with cytoplasmatic vesicles. These data provide initial evidence that NO synthesis in *R. farinacea* involves NR.

## 1. Introduction

Lichens are symbiogenetic organisms composed of fungi (mycobionts) and their photosynthetic partners (photobionts), which may be unicellular green algae (phycobionts, microalgae) or cyanobacteria [1,2] and bacterial communities. Lichens are nowadays in the focus of understanding multi-microbial symbioses evolutionary processes. They are poikilohydrous, subjected to repeated desiccation/rehydration cycles, and able to survive in extreme, frequently very dry environments, such as deserts or the arctic and Antarctic habitats. They can remain long periods with inactive metabolism and restart it again in the presence of water (reviewed by Kranner et al. [3]). Rehydration of lichens is a stressful situation that results in the massive release of reactive oxygen species (ROS). ROS are produced in the oxidative phosphorilation (respiratory) and photosynthetic electron chains, but their production increase during stress such as nutrient limitation and exposure to xenobiotics, and are a major cause of damage during desiccation-rehydration events, especially in photosynthetic organisms [4]. When desiccated, carbon fixation is limited by water deficiency, but electron flow continues, and excitation energy can be passed from photo-excited chlorophyll pigments to ground state oxygen, forming singlet oxygen (^1^O_2_). In addition, superoxides (O_2_^•−^), hydrogen peroxides (H_2_O_2_), and the hydroxyl radicals (^•^OH) can be produced at photosystem (PS) II [5]. If antioxidant defenses are overcome by ROS production, the uncontrolled free radicals cause widespread cellular damage by provoking protein alterations, lipid peroxidation, and the formation of DNA adducts [6]. The lichen symbiosis is intricately linked to desiccation tolerance, for which potent ROS scavenging machinery is essential [4].

Nitric oxide (NO) is an intra- and intercellular signaling molecule involved in the regulation of diverse biochemical and physiological processes. These functions include signal transduction, cell communication, stress signaling, and metabolism of free radicals (reviewed by Wilson et al. [7], Mur et al. [8]). NO has been postulated as one of the first protective mechanisms against ROS in eukaryotic cells [9]. It’s a dual functional molecule. While low levels of NO modulate the ROS such as superoxide anion [10,11], high concentrations of NO enhance superoxide production in mitochondria by inhibiting electron flow cytochrome *c* oxidase [12], producing peroxynitrite and causing lipid peroxidation and protein nitration. In the first case, modulation of superoxide formation and inhibition of lipid peroxidation by NO illustrates its less known potent antioxidant role [13,14]. Research on the role of NO in biological systems has increased since it was suggested in the latter part of the 1980s that it was an important signaling molecule in animals [15]. This function has also been studied in plants, bacteria [16] (reviewed by Gupta et al. [17]), algae [18,19,20], and fungi [21,22,23,24]. 

We have recently reported evidence that NO released during lichen *Ramalina farinacea* rehydration plays a fundamental role in the antioxidant defense and production appears to be regulated by ROS [25]. Regarding the phycobionts we have shown that they also generate significant quantities of NO, in contrast to the findings of Weissman and co-workers [26]. Moreover, our group has also demonstrated that NO is involved in the regulation of oxidative stress caused by exposure to the prooxidant air pollutant cumene hydroperoxide [27]. Although all these studies confirm the production of NO in *R. farinacea* and provide insight into its roles, no experimental designs have addressed the synthesis of NO in lichens or their symbionts.

In animal cells, biosynthesis of NO is primarily catalyzed by the enzyme NOS (reviewed by Wendehenne et al. [28]), that catalyzes the conversion of L-arginine to L-citrulline and NO using NADPH as electron donor, molecular oxygen as co-substrate, and FAD, FMN, tetrahydrobiopterin (BH_4_), and calmodulin (CaM) as cofactors [29]. Regarding plants, they are not only affected by the atmospheric pollutant NO, but they also possess the ability to produce NO by enzymatic and non-enzymatic pathways. Non-enzymatic NO formation can be the result of chemical reactions between nitrogen oxides and plant metabolites, nitrous oxide decomposition, or chemical reduction of nitrite (NO_2_^-^) at acidic pH (reviewed by Wendehenne et al. [28]). The first enzymatic source of NO to be identified in plants was the nitrate reductase (NR) [30]. This enzyme not only reduce nitrate to nitrite, it also catalyzes the reduction of nitrite to NO using molybdenum (Mo) as a cofactor and NADH or NADPH as an electron donor. Two isoforms of NR have been described in higher plants and eukaryotic algae: EC 1.6.6.1 is specific for NADH whereas EC 1.6.6.2 is able to use both NADH or NADPH [31]. Recently, ARC (Amidoxime Reducing Component) has been reported to catalyze NO production from nitrite taking electrons from NR in the microalga *Chlamydomonas,* allowing its synthesis in the presence of nitrate by means of a newly described NO-forming nitrite reductase activity [32]. In addition to NR as a possible source for NO, the existence of a mammalian-type NOS in plants has been under debate in recent years (reviewed by Wendehenne et al. [28,33]). Despite the intensive quest for NOS in vascular plants, the only NOS known in the Viridiplantae has recently been identified, cloned, purified, and characterized in the marine free living green microalga *Ostreococcus tauri* (Trebouxiophyceae) showing a 45% homology with human NOS [34]*.* The researchers have observed that *O. tauri* cultures in the exponential growth phase produce 3-fold more NOS-dependent NO than do those in the stationary phase and NO production increases in high intensity light irradiation.

In regard to the synthesis of NO in fungi there is little information, the evidence that there is a NOS associated to NO production are indirect and all rely on the use of inhibitors of this enzyme [23]. A specific fungal NR (EC 1.6.6.3) using NADPH as co-factor has been described [31]. 

NO is revealing itself as a keystone in stress tolerance of symbiotic associations such as *Symbiodinium*—cnidarian (corals), plant—*Rhizobium* or mycorrhizae, critical in global geomorphology and nitrogen ecology [35]. Thus, it is of the utmost interest to elucidate the mechanisms that mediate its production in lichens, symbiotic organisms inhabiting almost every terrestrial habitat. *R. farinacea* (L.) Ach is a fruticose lichen bearing in each thallus two predominant microalgae, *Trebouxia sp.* TR9 and *T. jamesii,* and a mycobiont belonging to the phylum Ascomycota [36]. We have previously demonstrated that NO limits intracellular free radical release and modulates lipid peroxidation during rehydration of these lichen thalli also protecting phycobiont chlorophyll from photooxidation [25,37].

The aim of this work is to gain insight into the synthesis of NO in the lichen model *R. farinacea.* To this end we have studied the effect of specific enzyme inhibitors on lipid peroxidation upon rehydration and a preliminary quantification of plant-like NR specific activity has been obtained.

## 2. Results

### 2.1. Effects of NR Inhibition on Lipid Peroxidation during Lichen Rehydration

Our group previously reported that NO is involved in intracellular free radical modulation and lipid peroxidation prevention during *R. farinacea* thalli rehydration [25]. In order to test whether NR is involved in the production of this NO, the inhibitor tungstate was added during thalli rehydration. The results of lipid peroxidation when lichen thalli were rehydrated with tungstate inhibitor are shown in Table 1. In the case of the controls, MDA concentration was between a minimum value at 5 min of 81.47 ± 8.14 nEq MDA/g lichen and a maximum of 131.41 ± 18.80 nEq MDA/g lichen at 120 min. In thalli rehydrated with tungstate 100 μM, MDA concentration was between a minimum value at 5 min of 83.98 ± 6.28 nEq MDA/g lichen and a maximum of 191.88 ± 11.06 nEq MDA/g lichen at 120 min. At all test times, treatment MDA levels were higher than controls with statistically significance at 120 min. 

**Figure 1:**
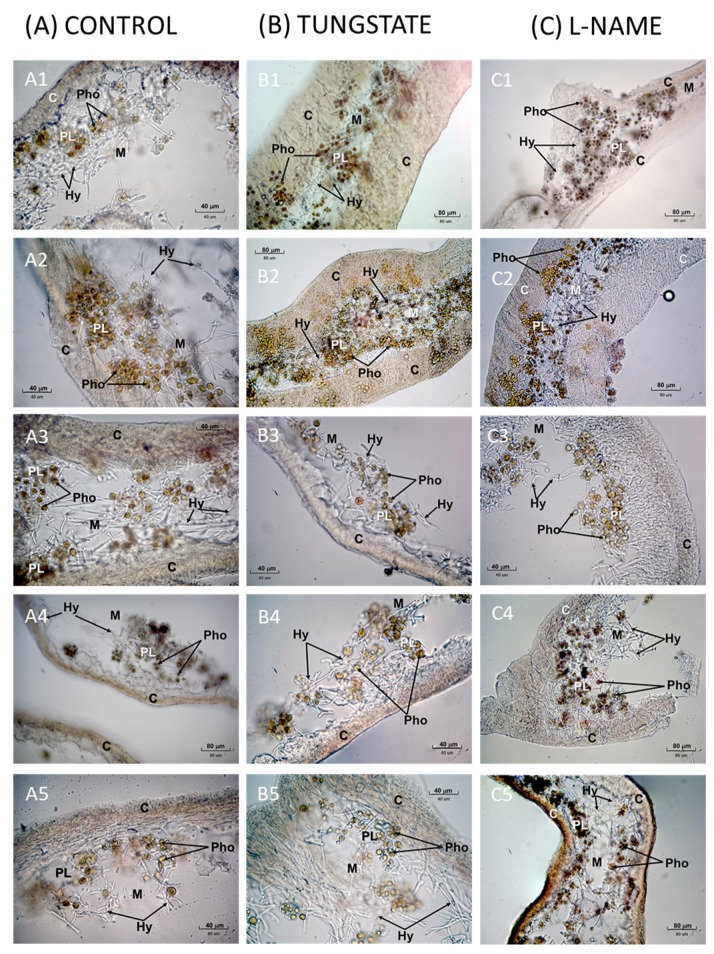
Bright field microscopic images of pink-brown TBARS in thalli of *R. farinacea* rehydrated with tungstate 100 µM (**B**), L-NAME 300 µM (**C**) vs. thalli rehydrated with deionized water (**A**). One representative image of different independent experiments has been selected for each condition. The number by the letter identifying the picture indicates the time post-rehydration when TBA reaction was revealed: (**1**) 5 min, (**2**) 30 min, (**3**) 60 min, (**4**) 120 min and (**5**) 240 min. Magnitude bars in the microphotographs correspond to 40 or 80 µm. C cortex with chondroid tissue, PL phycobiont layer, Pho microalgae, M medulla, Hy fungal hyphae.

Morphological distribution of lipid peroxidation in pink and brown tones is shown in Figure 1B where only one representative picture from replicated experiments has been selected. Despite microscopy is not a quantitative technique, at all time points, the coloration in the controls was less intense than in thalli rehydrated with tungstate. However, visual differences were only perceived at 5 (B1) and 30 (B2) minutes. There were not remarkable visual differences at 60 (B3), 120 (B4), and 240 (B5) minutes. In both cases, controls and thalli rehydrated with tungstate, lipid peroxidation was primarily located in the hyphae of the cortex and chondroid area and in the microalgae. In the hyphae of medulla, lipid peroxidation was lower. 

### 2.2. Effects of Nitric Oxide Synthase (NOS) Inhibition on Lipid Peroxidation during Lichen Rehydration

The results of lipid peroxidation when lichen thalli were rehydrated with L-NAME are shown in Table 2. In the case of the control, a maximum of 110.51 ± 12.17 nEq MDA/g lichen at 30 min was observed and a minimum value of 44.74 ± 4.66 nEq MDA/g lichen at 240 min. In the rehydration with L-NAME a maximum of 137.51 ± 11.77 nEq MDA/g lichen at 30 min was found and a minimal value of 74.56 ± 6.29 nEq MDA/g lichen at 240 min. MDA concentration in the treated thalli was always higher than in the controls. The differences are statistically significant at 120 and 240 min.

Morphological distribution of lipid peroxidation in pink and brown tones is shown in Figure 1C. Only one representative picture from the experimental replicates is shown. Lipid peroxidation in the hyphae of the chondroid cortical area and medulla was lower than in controls. At 5 (C1), 30 (C2), and 120 (C4) minutes, the microalgae of thalli rehydrated with L-NAME were more affected by lipid peroxidation than controls (see brown color in phycobionts). However, these thalli show lower lipid peroxidation in the hyphae of the cortical zones and medulla than controls. At 60 min (C3) lipid peroxidation appeared to be higher in the hyphae of the cortex and in the phycobionts of the controls (dark brown areas). Finally, at 240 (C5) minutes lipid peroxidation was greater in the thalli treated with the inhibitor than in controls and it was localized in the peripheral areas and in the microalgae (very dark areas). This was the time when the greatest visual differences were observed. 

### 2.3. NO Endproducts 

At all times, NOx levels of thalli rehydrated with tungstate were lower than controls (Table 3). NOx production in controls was between a minimum absolute value of 0.05 ± 0.01 μmol NOx/g lichen (DW) and a maximum of 0.26 ± 0.03 μmol NOx/g lichen (DW). NOx production in thalli rehydrated with tungstate was between a minimum absolute value of 0.03 ± 0.01 μmol NOx/g lichen (DW) and a maximum of 0.16 ± 0.03 μmol NOx/g lichen (DW). At 30 and 120 min, statistically significant differences were found.

NOx levels of lichen thalli rehydrated with L-NAME were greater than controls at all times, except for 5 min (Table 3). NOx production in controls was between a minimum absolute value of 0.008 ± 0.001 μmol NOx/g lichen (DW) and a maximum of 0.034 ± 0.005 μmol NOx/g lichen (DW). NOx production in thalli rehydrated with L-NAME was between a minimum absolute value of 0.012 ± 0.001 μmol NOx/g lichen (DW) and a maximum of 0.030 ± 0.006 μmol NOx/g lichen (DW). Statistically significant differences were found at 30, 120, and 240 min.

### 2.4. Diaphorase Activity

**Figure 2:**
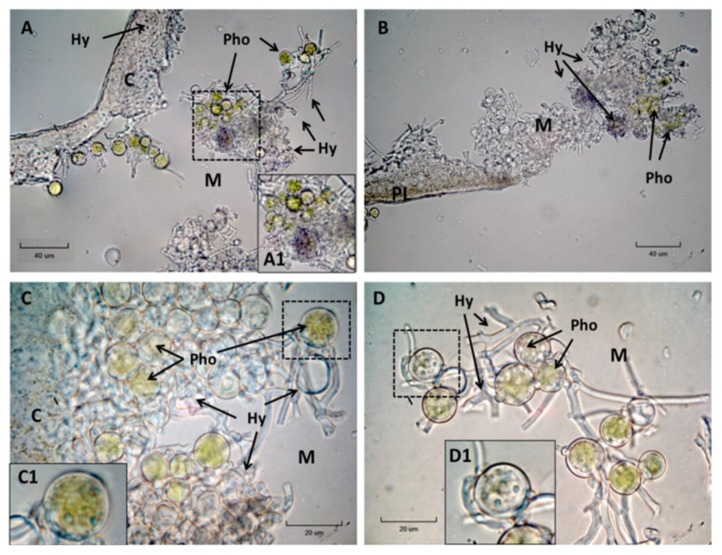
Diaphorase activity assayed with nitrotetrazolium blue chloride (NBT, blue precipitates) in *R. farinacea* thalli (**A**–**D**). Bright-field microscopy of slices cut in a freezing microtome (magnification 1000×). The areas framed with discontinuous lines have been digitally magnified in the corresponding insets (**A1**, **C1** and **D1**). C with chondroid tissue, PL phycobiont layer, Pho microalgae, M medulla, Hy fungal hyphae.

Histochemical detection of NADPH-diaphorase activity has been related with NOS in animal and plant tissues [38]. At 2 h (Figure 2A–D) blue precipitates were observed in the hyphae, both in the cortex and chondroid area, but especially in the latter. Small vesicles with blue precipitate were seen inside phycobionts (Figure 2(C1,D1)). In the peripheral zone of microalgae, blue precipitates were also found (Figure 2(A1)). Assuming that the NADPH-diaphorase activity represent the NOS-like activity, the results here suggest the occurrence of NOS-like enzymes in *R. farinacea.*

### 2.5. Specific Activity of NR

As other Chlorophyta, *Trebouxia* phycobionts of *R. farinacea* are likely to possess NADH-NR activity. Despite no method to assess NR activity has been reported for lichens to our knowledge, we used a general method for plants [39] in whole thalli in order to obtain a value of the specific activity of NADH-NR in *R. farinacea*. The value obtained for NADH-NR specific activity in fresh *R. farinacea* thalli was (0.91 ± 0.13) × 10^−4^ U/mg protein (U = µmoles nitrite/min). In order to check if tungstate was capable of inhibiting this measured activity, we used increasing concentrations of this inhibitor. The activity of NADH-NR decreased as the concentration of tungstate increased in a dose dependent manner and was not measurable above 50 µM of the inhibitor (Supplementary Figure S1).

## 3. Discussion

NO is revealing itself as a key molecule in the tolerance of abiotic stress of symbiotic organisms as mycorrhizae, *Rhizobium*, and lichens and the elucidation of the mechanisms and regulation of its synthesis will provide very valuable information both for conservation of biodiversity as well as for biotechnological purposes. The increase in lipid peroxidation upon the inhibition of NR and NOS-like activities in the model lichen *R. farinacea* described in the present work suggests the participation of both enzymes in the synthesis of NO under rehydration stress. 

In lichen thalli rehydrated with the NR inhibitor tungstate, lipid peroxidation increased compared to control, while NO release decreased as expected. Lipid peroxidation indirectly indicates that the antioxidant defenses have been overcome by the formation of reactive oxygen species (ROS) [6]. Although NO donors have been shown to reduce antioxidants, inhibit or inactivate antioxidant enzymes and increase MDA through H_2_O_2_ accumulation in stressed plants (reviewed by Groß et al. [40]) it has also been shown to decrease the generation of ROS and thus, lipid peroxidation in plant microsomes [13,14] and lichens [27,37]. As a matter of fact, it is able to directly terminate free radical chain reactions [41]. The use of tungstate as NR inhibitor has to be considered with caution because of side effects due to the affection of other molybdenum-enzymes or heavy metal toxicity, especially at longer exposure times [42]. However, this important result correlates with c-PTIO lichen NO scavenging [25] and points to the existence of a NO related NR activity in *R. farinacea.* This is the first study providing evidence that NR may be implicated in the synthesis of NO during abiotic stress in lichens or lichen symbionts. NR has also been involved in the synthesis of NO in the green microalga *Chlamydomonas reinhardtii* [43]. As a matter of fact, Mallick et al. [44] and Medina-Andrés et al. [45] propose that the synthesis of NO is a common feature for algae as well as embryophytes and is strongly dependent on NR.

A study with *Pleurotus eryngii var. tuoliensis*, a basidiomycete fungus, showed that heat stress induced an increase in NO production in mycelial cells which was significantly blocked by NOS inhibition (L-NAME). In contrast, NR activities were not obviously altered during heat stress [23]. But NO levels required in the morphogenesis and reproduction of the ascomycetes fungus *Aspergillus* seem to be insufficient without a functional NR gene [46]. *R. farinacea* mycobiont is an ascomycete and the gene for NR has been reported to be part of a cluster of genes that were transferred horizontally from a basidiomycete to an ancestor of the ascomycetes [47]. These data support the existence of a functional NR enzyme also in ascomycetes as a plausible hypothesis.

In thalli rehydrated with NOS inhibitor L-NAME, lipid peroxidation slightly increased in both symbionts but, unexpectedly, NOx endproducts increased too. This suggests that NO levels are higher in thalli rehydrated with L-NAME than in thalli rehydrated with deionized water but yet not efficient in lipid peroxidation prevention. This L-arginine analogue is a reversible inhibitor whose paradoxical ability to induce NO increases by NOS activity upregulation was reviewed by Kopincová et al. [48]. NOS enzymes have been demonstrated to be finely regulated both at protein and expression levels depending on the physiological conditions of the organism. NO chemistry is complex and its sources, multiple, which could generate local effects linked to spatial and morphological constraints to NO bioavailability and activity [48]. Although thallus NOS-like activity was inhibited, NO could still be synthesized by NR activity or by non-enzymatic pathways (reviewed by Wendehenne et al. [28]) resulting in overproduction. Accumulation of NO in response to stress has been associated with increased ROS levels due to inhibition of antioxidant enzymes and formation of peroxinitrites (reviewed by Gross et al. [49]). Despite quantitatively lower, NOS-like activity inhibited by L-NAME seems to be especially critical since its inhibition triggered an upregulation of other NO sources which, in turn, seem not to be efficient in lipid peroxidation limitation. Although unknown side effects of L-NAME in lichens cannot be ruled out, the development of a method for total NR activity quantification in lichens to test a possible upregulation during NOS activity inhibition, together with morphological localization of NO release would shed light on this paradox.

In the same line, cytochemical NADPH-diaphorase activity demonstration points to a NOS-like activity in *R. farinacea* analogue to animals and plants [38]. Diaphorase activity has been detected in the hyphae, both the cortical plectenchyma and medulla, in vesicles inside and in the periphery of the microalgae. In the marine microalga *Chattonella marina*, the main source of NO production has been reported to be NOS activity [50]. Recently, the first NOS in Viridiplantae has been identified and characterized by Foresi et al. [34] in the marine green microalga *Ostreococcus tauri*. Valentovicová et al. [51] showed that L-NAME inhibited both NADPH-diaphorase activity and NO production in barley root tips. However, NADH-diaphorase activity has been reported for NADH-NR and this activity cannot be ruled out for fungal NADPH-NR or other enzymes, further experiments are necessary to confirm the presence of a NOS enzyme.

Our data show that when NR is inhibited, lipid peroxidation is primarily located in the hyphae of the cortex and chondroid plectenchymas and in phycobionts, while when NOS is inhibited, lipid peroxidation increases in microalgae. As pointed out above, this means that the mechanisms and kinetics of synthesis of NO determine, at least in part, its role: While NR has an important role in the protection of both mycobiont hyphae and phycobionts in the first hours, a fungal NOS, sensitive to animal NOS inhibitors and immunoreactive with animal NOS antibodies, has been described both in ascomycetes and yeasts although, alike plants, gene orthologues have not been found and responsible proteins have not been characterized [52]. NOS-like activity inhibited by L-NAME seems critical for microalgae from the very first minutes after rehydration. We don´t know how many NR and NOS-like enzymes there might be in this lichen and if so, which participates in the synthesis of NO in stress conditions. We must bear in mind that *R. farinacea* contains, at least, three different eukaryotic organisms (fungi, yeasts, microalgae) from two supergroups (Opisthokonta and Archaeplastida) and each could possess its own NR and NOS enzymes with specific peculiarities in expression regulation, suborganellar localization, kinetics, or allosteric modulation. This provides symbiotic organisms with a versatile set of tools to cope with abiotic stress.

The value found for plant-like NADH-dependent NR specific activity in *R. farinacea* (0.91 × 10^-4^ U/mg) is two orders of magnitude lower than NR specific activity reported for the Chlorophyceae *Ulva intestinalis* (Table 4) [53]. A much more similar value of 0.40 × 10^−4^ U/mg has been reported for the bryophyte *Physcomitrella patens* [45]. The specific activities reported for various marine macroalgae of the Rhodophyta show some divergences, whereas in *Kappaphycus alvarezii* (Gigartinales), specific activity is 0.16 U/mg [54], *Gracilaria tenuistipitata* (Gracilariales) specific activities of NR reported for crude extracts are ten times higher (3.0 ± 0.2 in apical part, 1.6 ± 0.1 U/mg for basal) [55]. However, a more recent study characterizing *Gracilaria chilensis* by Chow et al. [56] has reported 2.53 × 10^−4^ U/mg, and a value of 8.33 × 10^−4^ U/mg has been obtained for *Porphyra perforata* (Bangiales) [53] comparable to *Arabidopsis thaliana*’s 2.50 × 10^−4^ U/mg [57]. In the same order of magnitude, NR specific activity reported for *Gracilaria caudata* is 0.93 × 10^−4^ U/mg [39] and for *Gracilaria tikvahiae* is 0.43 × 10^−4^ U/mg [58].

As symbiotic organisms, lichens are composed of algae, fungi, and bacteria. Given that for this preliminary approach we have used a NADH-method designed for plants, we are only taking into account the plant/algae isoforms, namely EC 1.6.6.1 and EC 1.6.6.2. The possible existence of a fungal isoform (EC1.6.6.3) specific for NADPH as co-factor remains to be elucidated. On the other hand, most of photosynthetic organisms seem to possess NADH-specific EC 1.6.6.1 isoform, but some microalgae have shown a small nitrate reducing activity with NADPH (EC 1.6.6.2) [59]. NR specific activity studies with the isolated microalgae (Trebouxiophyceae) of *R. farinacea* are necessary to rule out whether one or both isoforms are present. In any case, in the absence of fungal biomass we can reasonably expect higher values, likely in the range of the Chlorophyceae *Ulva intestinalis.*

Phycobionts are probably the main source of the specific activity we report using a plant-designed method with *R. farinacea* whole extract. However, we report above the induction of lipid peroxidation in fungal hyphae upon NR inhibition with tungstate. Since the mycobiont accounts for the greater part of the biomass of the thallus, a remarkable NR specific activity could also be expected if NADPH were used as co-factor. We are currently working on optimizing a method specifically designed for lichens.

Our approach has allowed us to demonstrate NR and NOS-like enzymes activities in *R. farinacea,* but the evidence of these enzymes is indirect and the presence of the proteins themselves should be further verified. The quantification of NADH-NR, although preliminary, adds to the evidences. Nonetheless, in order to confirm the presence of NOS-like in each symbiont and to characterized NR isoforms, future studies are required about the biosynthesis of NO in the microalgae as well as in the isolated *R. farinacea* mycobiont. Studies to isolate the proteins and genetic studies will also be necessary.

## 4. Materials and Methods 

### 4.1. Chemicals

2-Thiobarbituric acid (TBA), sodium tungstate dihydrate (Na_2_WO_4_•2H_2_O) and 1,1,3,3 tetraethoxypropane (TEP), nitrotetrazolium blue chloride (NTB), 2,6-di-tert-buthyl-4-methylphenol trichloroacetic acid (BHT), bovine serum albumin (BSA), L-Cysteine, sulfanilamide (C_6_H_8_N_2_O_2_S), N-1-(naphthyl) ethylenediamine dihydrochloride (C₁₂H₁₆Cl₂N₂), and NADPH were provided by Sigma Aldrich Química S.A (Tres Cantos, Spain); NG-Nitro-L-arginine methyl ester (L-NAME) was purchased from Sigma Aldrich (China); Ethylenediaminetetraacetic Acid (EDTA) and trichloroacetic acid (TCA) was from Merck (Germany); dithiothreitol (DTT) and NADH were from Roche Custom Biotech; inorganics and ethanol (etOH) were purchased from Panreac Quimica S.A.U (Spain); triton X-100 was from VWR Prolabo (Barcelona, Spain).

### 4.2. Lichen Material

*R. farinacea* (L.) Ach. lichen thalli were collected in the air-dry state from *Quercus pyrenaica* in San Lorenzo de El Escorial at 969 m altitude (Ermita Virgen de Gracia, Madrid, Spain; 40°34′25,6′′ N, 4°9′146′′ W). Samples were maintained in a silica gel atmosphere during 24 h and frozen at −20 °C until the experiment.

### 4.3. Treatments

Lichen thalli were cut and weighed between 20–30 mg. For each time point (0, 30, 60, 120, and 240 min) 12 replicates were processed with each inhibitor (100 µM sodium tungstate dihydrate or 300 µM L-NAME) and controls. The day of the experiment, fragments of lichen thalli were rehydrated during 5 min with deionized water for controls or one of the inhibitors. Then, they were maintained at room temperature for the times of study and subsequently frozen at −20 °C until lipid peroxidation analysis. Inhibitor concentrations were selected according to the literature regarding plant NR [42] and fungal NOS [23].

### 4.4. Measurement of Lipid Peroxidation: MDA

The most common method for measuring MDA in biological samples is the thiobarbituric acid reactive substances (TBARS), which is based on spectrophotometric quantification of the pink complex formed after reaction of MDA with two molecules of TBA [60] with maximum absorbance at 532 nm [61]. In our study, lipid peroxidation was evaluated as MDA by a variant of the method of Reilly and Aust [62]. As standards, 0–20 µM TEP were used as an MDA precursor. The reaction of TEP in acid medium generates the same complex TBA-MDA-TBA, allowing to relate the absorbance with the concentration of the complex. The presentation of the results of lipid peroxidation will expressed as nEq MDA/g of sample, as a measure of the amount of MDA in the sample.

Lichen thalli were homogenized with 1 mL of deionized water on ice and centrifuged at 16,060× *g* for 10 min to eliminate debris. Supernatants were frozen at −20°C for NO_x_ analysis and sediments were resuspended in 500 µL ethanol—BHT 2%. A volume of 900 µL of TBA (2.57·10^−2^ M), TCA (9.18·10^−1^ M) and HCl (3.20 M) working solution was added to each sample and standard. Then, samples and standards were vortexed in a Vortex Labnet × 100 for 5 min at 3000 rpm and placed in a water bath at 70°C for 30 min. Next, samples and standards were vortexed, cooled in ice and centrifuged 10 min at 16,060× *g*. Finally, absorbance of the supernatants from samples and standards was measured at 532 nm and 600 nm to eliminate the interferences of soluble sugars in samples, in a spectrophotometer Anthos 2010, model 17-550.

To analyze the morphological distribution of lipid peroxidation, fragments of treated lichen thalli were subjected directly to TBARS method (described above), but they were not homogenized. Then they were placed in a freezing microtome (CM 1325; Leica, Germany) and sliced into sections of 30 µm. The slices were washed with deionized water and mounted on slides prior to their observation by optical microscopy (OLYMPUS Provis AX 70 optical microscope) equipped with an infinity 2-3C Lumenera^®^ digital camera and analysed with “Infinity Analyze” Software v.6.5.5 at the Plant Anatomy Laboratory “Julio Iranzo” in the Botanical Garden of the University of Valencia.

### 4.5. NO Endproducts Determination

The products formed by the oxidation of NO in an aqueous environment are mainly NO_2_^−^, which is further oxidized to NO_3_^−^ [63]. In order to estimate NO generation, NO oxidation endproducts (nitrates and nitrites) were measured in the soluble fraction of different samples with an autoanalyzer Skalar, model SAN++. The automated determination of nitrates and nitrites is divided in two phases: first, the reduction of nitrates to nitrites by the cadmium reduction method, where the sample is passed through a column containing granulated copper-cadmium; second phase involves the reaction of nitrites with N-(1-naphthyl) ethylenediamine dihydrochloride in acid medium to form a highly coloured azo dye which is measured at 540 nm. This method is known as Griess reaction [64,65].

### 4.6. Diaphorase Activity

The basic protocol used to detect diaphorase activity in animal neurons [66,67] was used in a modified manner in this study. Diaphorase activity was observed after 2 h. During these times, lichen thalli were incubated in a solution of 0.5 mg/mL NADPH and 0.2 mg/mL NBT in phosphate buffered saline (PBS) with 0.25% of Triton X-100. Then, thalli were washed three times with deionized water and frozen at –20 °C. The samples were then placed in a freezing microtome (CM 1325; Leica, Germany) and cut in sections of 30 µm. The slices were washed with deionized water and mounted on slides prior to their observation by optical microscopy (OLYMPUS Provis AX 70 optical microscope) at the Jardí Botànic and SCSIE (UVEG, Valencia).

### 4.7. Specific Activity of NR

The enzymatic assay of NR was performed as described in [39] with minor changes. Samples of lichen thalli of *R. farinacea* (250 mg) were milled in a conical homogenizer and suspended in 5 mL of extraction buffer (1 mM DTT, 5 mM EDTA, 1 mM cysteine, 0.3% BSA (w/v), and 0.2 M phosphate buffer, pH = 7.5) to stabilize NR. Cell debris was removed by centrifugation at 17000 g for 15 min at 4 °C. An aliquot of the supernatant was taken for total soluble protein quantification. The supernatant (crude extract) was recovered and kept on ice until the activity of the enzyme was analyzed. To 100 μL of crude extract 20 μL of KNO_3_ 500 mM, 20 μL of MgSO_4_ 9.5 mM, and 50 µL of NADH 380 µM were added to initiate the reaction. The reaction was interrupted after 10 min by adding 20 μL of ZnSO_4_ 1.4 mM and 20 μL of cold ethanol 90% v/v. The precipitates were removed by centrifugation at 12,000 *g* for 10 min at 20 °C and the Griess method [68] was used to analyze nitrite production as described in Chaki et al. [69] although some changes were applied. To 190 μL of sample 95 μL of 1% sulfanilamide (w/v) in 1.5 M HCl and then 95 μL of 0.2% n-naphthylethylenediamine (w/v) in 0.2 M phosphate buffer pH = 7.5 were added. A measurable azo dye at 540 nm was developed after 5 min. NaNO_2_ was used as a standard in a range between 0–10,000 μg/L. Nitrate blanks were performed to account for non-enzymatic nitrite content of lichen samples. Bradford method [70] with some modification was used to quantify total soluble protein: 5 μL of sample were mixed with 250 μL of Bradford reagent, and absorbance was measured at 595 nm after 10 min. A standard curve was made with concentrations ranging from 0 to 1 mg BSA/ml extraction buffer. Blanks without the substrates were performed with each sample analyzed. The activity value obtained in the absence of these substrates informed about natural levels of nitrite in thalli. Nitrate blank was the highest and was subtracted from total activity to account for non-enzymatic nitrite. NR activity units (U) were defined as μmoles nitrite produced/min at 25 °C and pH 7.5.

### 4.8. Statistics

For each treatment and study times at least 12 replicates were prepared (n = 12). The results are expressed as means ± standard error. Software used for statistical analysis was “R-commander”. We determined the significant differences between treatments in each time using a Student’s *t*-test when the variances were equal, and the Welch’s test when the variances were different. Comparison of variances was made with a statistical test based on the distribution F of Snedecor. Before statistical analysis, the normality of the data was verified by the application of Shapiro–Wilk test. in all cases was considered significant a *p*-value < 0.05. For the quantification assay of NR activity, 3 replicates were used. The results are expressed as means ± standard error.

## 5. Conclusions

Our results demonstrate the existence of NR activity correlated with NO generation in the lichen *R. farinacea* under stress conditions. NO role seems to be determined by its source: NO related to NR activity seems to have an important role in the hyphae of cortex and in phycobionts in the first hours, while NO correlated with NOS-like seems to be more important for microalgae. NADPH-diaphorase activity cytochemistry supports the existence of NOS-like activity in both the mycobiont and the phycobionts of *R. farinacea*, where it is associated with big cytoplasmatic vesicles. Preliminary quantification of NADH-NR specific activity has rendered 91.00 ± 13.23 µU/mg. Taken together these data indicate that NO regulation and synthesis in lichens is complex involving both NR and NOS-like activities.

## Figures and Tables

**Table 1:** Effect of tungstate on the lipid peroxidation level in differently rehydrated *R. farinacea* thalli. * *p* < 0.05.

Time of Rehydration (min)	Lipid Peroxidation Level (nEq MDA/g Dry Weight)		*p* Value (Student’s *t*-Test)
	Control	100 µM Tungstate	
5	81.47 ± 8.14	83.98 ± 6.28	0.809
30	102.21 ± 12.43	115.16 ± 7.42	0.381
60	113.70 ± 13.73	144.82 ± 18.42	0.189
120	131.41 ± 18.80	191.88 ± 11.06	0.011 *
240	87.69 ± 7.61	108.60 ± 7.36	0.061

**Table 2:** Effect of L-NAME on the lipid peroxidation level in differently rehydrated *R. farinacea* thalli. * *p* < 0.05.

Time of Rehydration (min)	Lipid Peroxidation Level (nEq MDA/g Dry Weight)		*p* Value (Student’s *t*-Test)
	Control	300 µM L-NAME	
5	81.47 ± 8.14	110.86 ± 14.90	0.09741
30	110.51 ± 12.17	137.51 ± 11.77	0.12500
60	104.32 ± 11.76	121.60 ± 10.29	0.28040
120	72.77 ± 5.46	89.45 ± 4.69	0.03021 *
240	44.74 ± 4.66	74.56 ± 6.29	0.00096 *

**Table 3:** Effect of tungstate and L-NAME on NO endproducts levels in differently rehydrated *R. farinacea* thalli. *p* value was calculated by Student’s *t*-test. * *p* < 0.05.

Time of Rehydration (min)	NO Endproducts Levels (% Relative to Controls)			
	100 µM Tungstate	*p* Value	300 µM L-NAME	*p* Value
5	72.70 % ± 19.23 %	0.3496	88.77 % ± 16.45 %	0.4554
30	28.11 % ± 4.20 %	0.0018 *	188.56 % ± 24.96 %	0.0038 *
60	67.22 % ± 14.34 %	0.2946	128.40 % ± 18.73 %	0.2369
120	55.65 % ± 11.11 %	0.0077 *	235.78 % ± 41.25 %	0.0079 *
240	73.62 % ± 22.93 %	0.4206	143.39 % ± 11.59 %	0.0439 *

**Table 4:** Specific NR activities referred to total soluble protein.

Species	Taxonomic Group	NR Specific Activity (U = µmol Nitrite/min)
*Ramalina farinacea*	Lichen (Chlorophyta − Trebouxiophyceae + Ascomycota)	(0.91 ± 0.13) × 10^−4^ U/mg present work
*Ulva intestinalis*	Chlorophyta − Chlorophyceae	0.27 × 10^−2^ U/mg [53]
*Physcomitrella patens*	Bryophyta	0.40 × 10^−4^ U/mg [45]
*Porphyra perforata*	Rhodophyta, Bangiophyceae	8.33 × 10^−4^ U/mg [53]
*Kappaphycus alvarezii*	Rhodophyta, Gigartinales	0.16 U/mg [54]
*Gracilaria tenuistipitata*	Rhodophyta, Gracilariales	3.00 ± 0.20 (apical) U/mg [55] 1.60 ± 0.10 (basal) U/mg [55]
*Gracilaria tikvahiae*	Rhodophyta, Gracilariales	0.43 × 10^−4^ U/mg [58]
*Gracilaria chilensis*	Rhodophyta, Gracilariales	(2.53 ± 0.03) × 10^−4^ U/mg [56]
*Gracilaria caudata*	Rhodophyta, Gracilariales	0.93 × 10^−4^ U/mg [39]
*Arabidopsis thaliana*	Magnoliophyta	2.50 × 10^−4^ U /mg [57]

## Supplementary Materials

The following are available online at https://www.mdpi.com/2223-7747/8/7/189/s1, Figure S1: Tungstate inhibition of lichen *R. farinacea* nitrate reductase activity.

## Abbreviations

BHT	2,6-Di-tert-buthyl-4-methylphenol
BSA	Bovine serum albumin
DTT	Dithiothreitol
DW	Dry weight
etOH	Ethanol
EDTA	Ethylenediaminetetraacetic Acid
L-NAME	NG-nitro-L-arginine methyl ester
MDA	Malondialdehyde
NBT	Nitro blue tetrazolium
NOx	Nitric oxide oxidation end-products
NOS	Nitric oxide synthase
NR	Nitrate reductase
PBS	Phosphate buffered saline
ROS	Reactive oxygen species
TBA	2-Thiobarbituric acid
TBARS	Thiobarbituric Acid Reactive Substances
TCA	Trichloroacetic acid
TEP	1,1,3,3- Tetraexthoxypropane
