# Supplementary figures and images for: Inhibition of NO Biosynthetic Activities during Rehydration of Ramalina farinacea Lichen Thalli Provokes Increases in Lipid Peroxidation
Source: Plants (Basel). 2019 Jun 26;8(7):189. doi: 10.3390/plants8070189 (PMC6681199; doi:10.3390/plants8070189)

## Slide 1
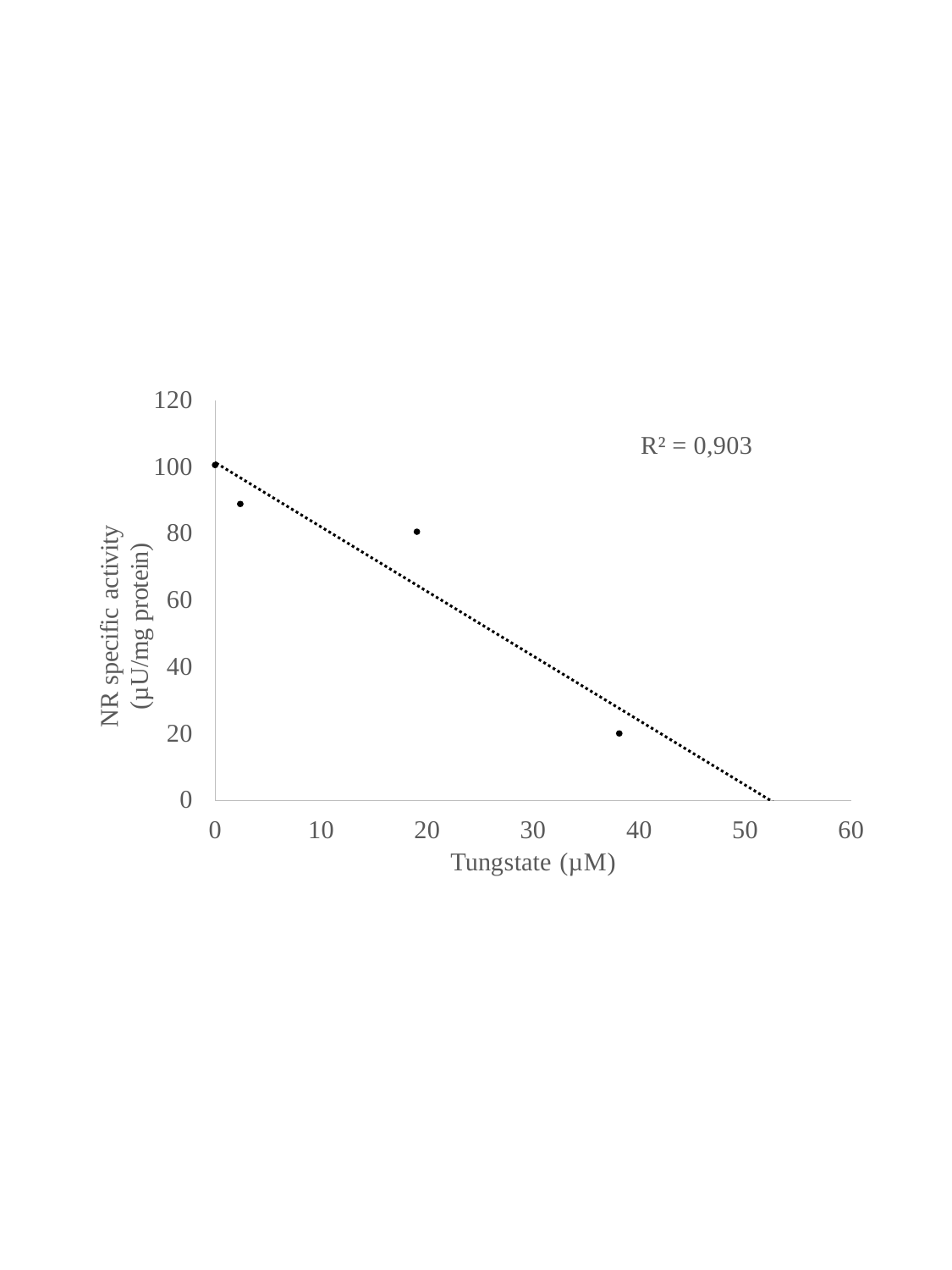

Supplement: Supplementary file 1 [file plants-08-00189-s001.zip › plants-486126-supplementary.pptx]
